# A consensus approach: Understanding the support needs of women in Newport West, Wales, to participate in breast screening

**DOI:** 10.1111/hex.13720

**Published:** 2023-02-09

**Authors:** Juping Yu, Sarah Wallace, Joyce Kenkre

**Affiliations:** ^1^ Faculty of Life Sciences and Education University of South Wales Pontypridd Wales UK

**Keywords:** breast screening, ethnic minorities, Group Concept Mapping, support, uptake of breast screening, Wales

## Abstract

**Introduction:**

Breast screening is an effective way to improve the early detection of breast cancer and reduce mortality. Unfortunately, low uptake of screening is often reported. This study aimed to explore the support needs of women residing in Newport West, Wales, to participate in breast screening.

**Methods:**

Group Concept Mapping, a structured participatory consensus approach, was used as the method. Participants completed three activities either online or offline: brainstorming to generate statements, sorting statements into themed categories; rating statements for perceived importance and accessibility (easy to get).

**Results:**

Thirty‐seven participants from seven ethnic groups took part. Sixty‐three statements (items of support) were generated and sorted into seven conceptually similar clusters (themes) (Trusting that I will be respected; Reassurance about my experience; Accessibility and convenience; Practical support; Addressing cultural diversity; Information tailored to individual needs; Raising awareness and understanding of breast screening). The ‘Trusting that I will be respected’ cluster was rated most important, while the ‘Practical support’ cluster was rated least accessible. Some disparity between responses was found based on ethnicity, language, disability and previous attendance of breast screening.

**Conclusions:**

Women require a range of support to participate in breast screening. The results highlight the importance of ensuring women feel and are respected, instilling trust in the staff performing the screening, offering reassurance about positive experiences of breast screening and providing practical support, especially individualized/targeted support for people who do not speak and/or read English and those with a disability.

**Patient or Public Contribution:**

The public contributed to the development of the information sheet, consent form, recruitment and data collection method.

## 1 INTRODUCTION

Breast cancer is a major threat to health and the most prevalent cancer globally, with 2.3 million new cases and 685,000 deaths associated with the disease in 2020.^1^ Its prevalence, mortality and survival rate differs across countries.^2^ In the United Kingdom, breast cancer is the most commonly diagnosed cancer, with around 55,920 new cases and 11,499 deaths each year.^3^ Most importantly, treatment for breast cancer can be highly effective, especially when the cancer is detected early.^1^

Breast screening is an effective way to identify people at risk of breast cancer, so that appropriate tests and treatment can be offered to promote better health outcomes.^4^, ^5^ Infrastructure and resources for free routine breast screening are in place in many developed countries.^6^ For example, administered by Breast Test Wales, the National Health Service Breast Screening Programme in Wales offers a population‐based service with mammography free of charge to all female residents aged 50–70 every 3 years.^7^

Not all invited women will attend breast screening, while late‐stage breast cancer and increased breast cancer mortality have been found to be associated with failure to attend screening.^8^, ^9^, ^10^ Breast screening‐related behaviour is very complex and has been explored widely in the United Kingdom and elsewhere. A wide range of factors have been identified, including personal (age, English speaking; ethnicity), socioeconomic (income; insurance), psychological (anxiety), cognitive (attitudes; beliefs; knowledge; intellectual capacity) and structural or organizational barriers.^11^, ^12^, ^13^, ^14^, ^15^, ^16^ Compared to the general population, lower breast screening attendance rates and breast cancer survival rates have been found in women within social and cultural groups in Wales and elsewhere, such as women from minority ethnic groups, with disabilities and from socially deprived areas.^17^, ^18^, ^19^

In Wales, primary care is organized into seven primary care clusters to bring together all local health and care services across a geographical area. Each primary care cluster is divided into several general practitioners (GPs) clusters (a group of GPs working with other healthcare professionals to deliver primary care services to a local population).^20^ The minimum target rate that each GP cluster is required to achieve for the uptake of routine breast screening invitations in Wales is 70%.^21^ The overall uptake rate in Wales was 69.1% in 2018–2019 before the outbreak of Covid‐19, which varied across GP clusters (53.9%–77.7%).^22^ The lower rates of uptake in some clusters may be reflective of the characteristics of populations in terms of ethnicity, disability and deprivation.^21^, ^22^ Aneurin Bevan University Health Board is 1 of the 7 primary care clusters, and the Newport West Neighbourhood Care Network is 1 of 12 GP clusters within the Health Board. It consists of eight GP practices providing primary care for people in Newport West. Newport is a multicultural city with the second largest proportion of ethnic minorities (12.5%) and is among the most deprived areas in Wales.^23^ Breast screening acceptance rate in this cluster was 65% in 2018–2019, which is below the target rate of 70%,^22^ Meeting this target is, therefore, a priority for the cluster.

Exploring barriers to participating in breast screening is important, as found in previous research conducted in many countries.^11^, ^12^, ^13^, ^14^, ^15^, ^16^ However, little is known about what support women really require to overcome these barriers and facilitate their participation in breast screening, especially in Wales. Enhancing knowledge in this area would provide valuable evidence to facilitate the development of interventions to increase uptake. In an early study carried out in Wales, Bell et al.^17^ examined the effects of interventions in increasing breast screening uptake in three general practices with a high proportion of patients from ethnic minority groups in Cardiff (Capital city of Wales). The interventions narrowly focused on addressing language issues and an endorsement letter from GPs. Interventions to increase uptake in other UK nations have included a nurse visit, a personal letter from general practices, telephone reminders/support, transport arrangements, language support, increasing awareness of breast cancer and improving attitudes towards breast screening.^24^, ^25^, ^26^, ^27^ However, there is a lack of evidence underpinning such interventions.

Considering socioeconomic and ethnic variations in breast cancer screening uptake in Wales and many countries, inventions to improve uptake (like any other interventions) need to be developed in a local context with its target population to address their needs.^28^ An important initial step is to understand what support is required from the perspectives of the target population.

## 2 METHODS

### 2.1 Aim

Commissioned by Newport West Neighbourhood Care Network in Wales, this study aimed to understand the support needs of women residing in Newport West, Wales, to participate in breast screening.

### 2.2 Design

Group Concept Mapping (GCM), a structured consensus‐building approach, was used as the method via Concept Systems GroupWisdom™ software.^29^ GCM is a participatory mixed‐methods approach, where qualitative elements (item generation, sorting, labelling and rating) are transformed into quantitative data as an integral process by speciality software to gain consensus on a specific topic of interest from a range of participants. The consensus emerges from the data via multidimensional scaling and hierarchical cluster analysis.^29^ Unlike other commonly used consensus methods (e.g., nominal group or Delphi techniques) that use several rounds of workshops or email conversations, only one round of data structuring is involved where participants independently sort statements, label each cluster of statements and rate statements to avoid groupthink or peer pressure. Furthermore, the results are co‐created by all participants and presented visually in a series of quantitatively derived concept maps and other reports to display ideas relevant to a given topic and how they are conceptually linked to each other, for example, which ideas are more or less important or relevant.^29^ GCM has been applied to many fields and contexts, including social and behavioural research, for example, to explore the concept of family resilience,^30^ the role of social prescribers,^31^ the concept of social well‐being^32^ and the complexities of managing children's care.^33^

A study steering group was established from the outset, drawn from nine professionals from Public Health Wales, primary care and the research team. The group reviewed and agreed on the focus prompt, rating scales, cluster solutions and cluster labels.

### 2.3 Participants and recruitment

The recruitment took place between June and October 2021. Eligible criteria for participation included women who were aged between 50 and 70, had been offered breast screening, had the capacity to give consent and resided in Newport West, Wales.

A recruitment poster was circulated via emails and social media (e.g., Facebook and Twitter) through community networks and the eight GP practices in the areas. Hard copies of the poster were on display in GP practices, local libraries and community centres in the area. GPs were not involved in the recruitment process. Participants contacted the researchers directly if they were interested to take part.

There is no strict limit on the number of participants in a GCM study, typically 40 or fewer with a minimum of 10, to ensure rich data while still manageable.^29^

### 2.4 Data collection (GCM procedure)

Participants completed three facilitator‐led activities (brainstorming, sorting and rating) over 4 months in 2021 either online via Concept Systems GroupWisdom™ software or offline by completing the required paperwork. Participants took part in one, two or all three activities based on when they were recruited and their willingness to participate.

The original plan was to collect data online only due to Covid restrictions. However, it was found that some participants experienced issues with completing the requested activities online, particularly older people and those who spoke little or no English. Therefore, an offline approach was adapted for data collection. There was no difference in the type of data collected by the two differing modes of participation.

For online participation, participants either completed the activities independently or with support from the first author over the telephone. For offline participation, relevant paperwork was posted to participants or co‐researchers, who were recruited and trained to recruit and collect data from people in ethnic minority communities. Participants completed the requested paperwork on their own or with support from a co‐researcher. When collecting data from participants who did not speak English, a co‐researcher translated the study information orally, and any data collected were translated into English by co‐researchers. The same type of data was collected from participants who completed the paperwork alone and those who completed it with support from a co‐researcher.

In addition, five demographic questions were asked (ethnicity; language; disability; screening uptake before March 2020; area of residence).

#### 2.4.1 Activity 1: Brainstorming (June–July 2021)

During the brainstorming activity (also known as ‘item generation’), participants generated as many statements (items of support) as they thought appropriate in response to the focus prompt: ‘Something that would help me to go for breast screening is…’. The activity was open for 5 weeks. For online participation, an invitation was emailed to participants, which contained the link to the study site, a unique login code, and instructions on how to take part. For offline participation, a sheet that contained the focus prompt and instructions on participation was provided.

The Key Words in Context method was used to review the raw statements generated by participants.^34^ The process involved reviewing the raw list, removing duplicates, splitting compound statements and checking for grammar and spelling mistakes. For example, the statement ‘Not mistreated or judged due to my background or language barrier’ was separated into two statements ‘Not feeling mistreated or judged due to my background’ (statement 4) and ‘Not feeling mistreated or judged due to my language barrier’ (statement 5). Additional statements identified from the literature were added to the list, which was reviewed first by the authors, followed by the study steering group. A final list of statements was agreed upon by the group in preparation for the sorting and rating activities. Kane and Trochim^29^ recommended limiting the final set of statements to 100 or fewer, which would provide a sufficient breath of representation of generated ideas while ensuring the sorting and rating activities were more manageable for participants to complete.

#### 2.4.2 Activity 2: Sorting (August–October 2021)

Participants sorted the final list of statements (items of support) by grouping them into categories that made sense to them based on perceived similarity and then labelled each category. The sorting activity (Activity 2) and the rating activity (Activity 3) took place in parallel for 8 weeks. For online participation, a drag‐and‐drop interface was available to sort statements into piles. For offline participation, participants were presented a set of individual cards with one statement per card and were asked to sort the cards into piles. Information about the statement numbers in each pile and pile names was recorded.

#### 2.4.3 Activity 3: Rating (August–October 2021)

Participants rated the final list of statements against two 5‐point Likert scales: importance and accessibility (easy to get) (1 = unimportant/not easy; 2 = somewhat important/easy; 3 = moderately important/easy; 4 = very important/easy; 5 = extremely important/easy).

*Rating scale 1: How important is this help?*

*Rating scale 2: How easy is it to get this help?*

### 2.5 Data analysis

Data analysis was conducted using Concept Systems GroupWisdom™ software. Data collected online were captured by the software automatically, while data collected offline were entered manually. Four steps were taken for analysis:

1. Responses to the five demographic questions were analysed using descriptive statistics.
2. A similarity matrix was created using sorted statements to show the frequency of how many times statements were sorted together into the same pile.
3. Multidimensional‐scaling analysis of the similarity matrix was carried out to produce a point map, where each statement of support was allocated a point on a two‐dimension (XY) axis.
4. Hierarchical cluster analysis was conducted to produce a series of diagrams and reports (a cluster map, cluster rating maps, pattern match reports and a Go‐zone report).

Eleven cluster solutions (4–15 clusters) were generated by the software. The authors reviewed the options first, and two solutions (6‐cluster and 7‐cluster solutions) were presented to the study steering group for feedback. A solution of seven clusters was chosen, based on what was considered to best reflect how participants grouped statements and the suggestion that the final solution should be determined by context and practicality.^29^

### 2.6 Ethical considerations

General ethical principles as set in the UK policy framework for health and social care research were followed.^35^

A standard version and an easy‐read version of the information sheet and consent form were provided to all prospective participants. Informed consent was sought from all participants. Participants were informed of their right to withdraw without any consequences and were assured that any data they provided would remain confidential and unidentifiable in any reports.

## 3 RESULTS

### 3.1 Participants

Thirty‐seven participants representing seven ethnic groups were recruited. Of these, 31 completed the brainstorming, 23 completed the sorting activity and 33 completed the rating activity. Table 1 shows the participants' demographics. The largest proportion of participants described themselves as White (*n* = 15), followed by Pakistani (*n* = 12). In addition to English, five other languages were spoken (Arabic, Bengali, Punjabi, Urdu and Italian). Twelve participants did not speak English.

**Table 1:** Characteristics of the participants.

Variables	Grouping	Frequency	Percent
Ethnicity	White	15	40.54
	Pakistani	12	32.43
	Arab	4	10.81
	Bangladeshi	3	8.11
	Black	1	2.70
	Indian	1	2.70
	Mixed or multiple ethnic groups	1	2.70
Disability	I have no disability.	26	70.30
	I have a long‐standing illness or health condition such as cancer, HIV, diabetes, chronic heart disease or epilepsy.	4	10.80
	I have physical impairment or mobility issues, such as difficulty using your arms or using a wheelchair or crutches.	3	8.10
	I have a disability, impairment or medical condition that is not listed above.	3	8.10
	I have a mental health condition, such as depression, schizophrenia or anxiety disorder.	2	5.40
Missed screening before March 2020	Never	24	64.86
	Once	7	18.92
	Twice	4	10.81
	Three times	2	5.41

Twenty participants reported that they had no disability, but others indicated that they had a long‐standing illness or health condition (*n* = 4), physical impairment or mobility issues (*n* = 3), a mental health condition (*n* = 3) or other types of disability, impairment or medical condition (*n* = 3). Three participants reported having two or more impairments and/or disabling medical conditions. Twenty‐four participants had never missed screening before March 2020, while 13 participants had missed screening once (*n* = 7), twice (*n* = 4) or three times (*n* = 2).

### 3.2 Brainstorming: Generating statements

During the brainstorming phase, participants (*n* = 31) generated an initial pool of 81 statements (items of support) in response to the focus prompt, ‘Something that would help me to go for breast screening is…’. After the cleaning process by the authors, the study steering group reviewed and agree on the final list of 63 statements (52 from participants; 11 from literature).

**Figure 1:**
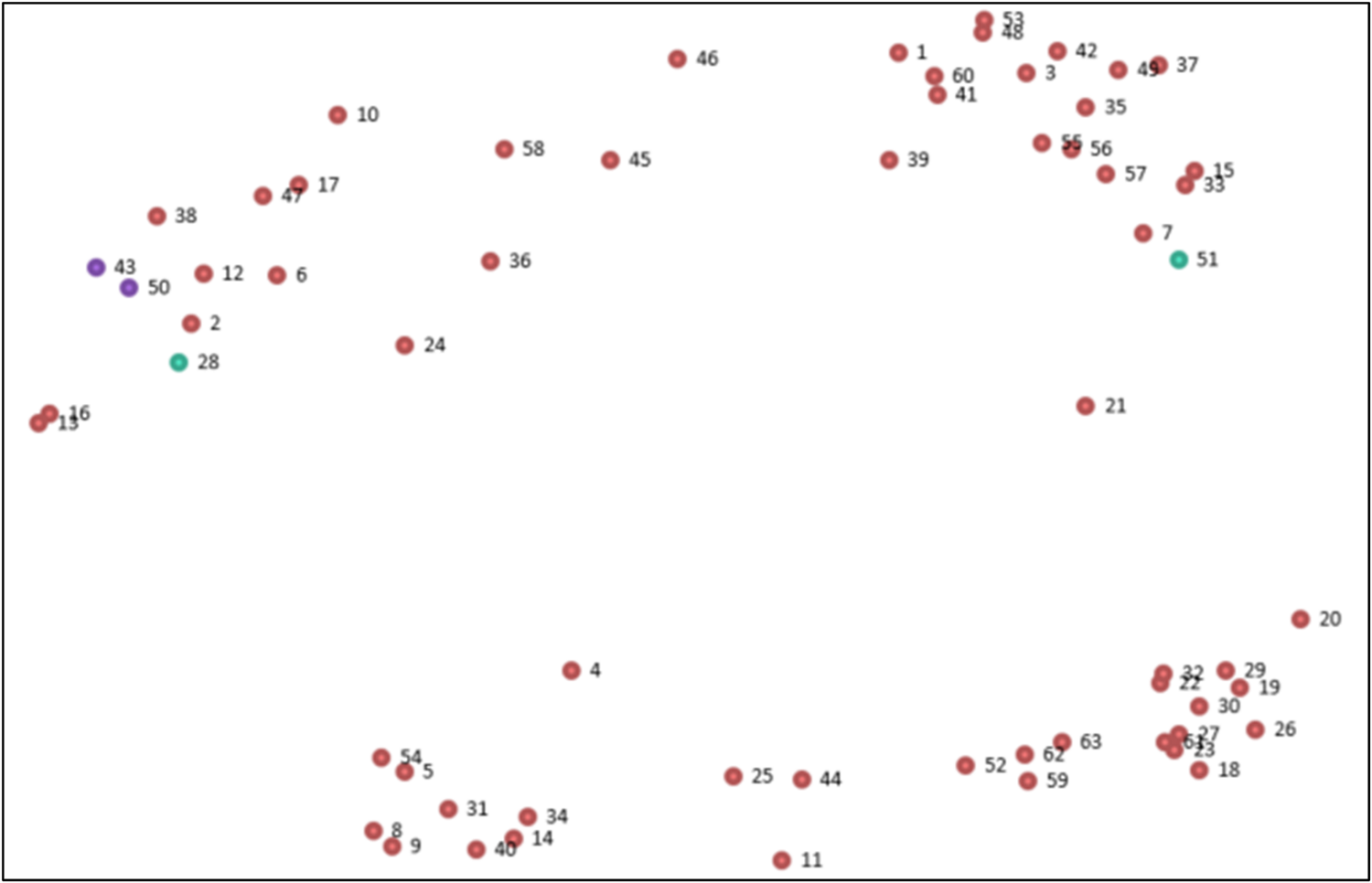
Point map of 63 statements (each point represents a statement accompanied by corresponding statement number).

All resulting statements (63) were used to populate a point map plotting statements on an XY graph (two‐dimensional solution using a multidimensional similarity matrix) based on how often statements were sorted together (Figure 1). There are 63 points on the map, and each point represents a statement of support. The distance between two points indicates how frequently the statements were sorted together. For example, statements 43 and 50 (in purple) are close together, as on average, participants sorted them together more frequently than with other statements that are further apart on the map. Conversely, statements 28 and statement 51 (in green) are very far apart, as they were not sorted together frequently or not at all.

The data set had a stress value of 0.20 after 11 iterations. The stress value is the key diagnostic statistic (similar to validity) in multidimensional scaling, which measures the degree of disparity between the distances on the map and the inputted similarity matrix data.^29^ The acceptable range is between 0.10 and 0.35, with a lower value indicating a better overall fit.^29^, ^34^ The stress value of this study is within the recommended range, implying a good relationship between the inputted data and the distances presented on the map.

### 3.3 Cluster maps

**Figure 2:**
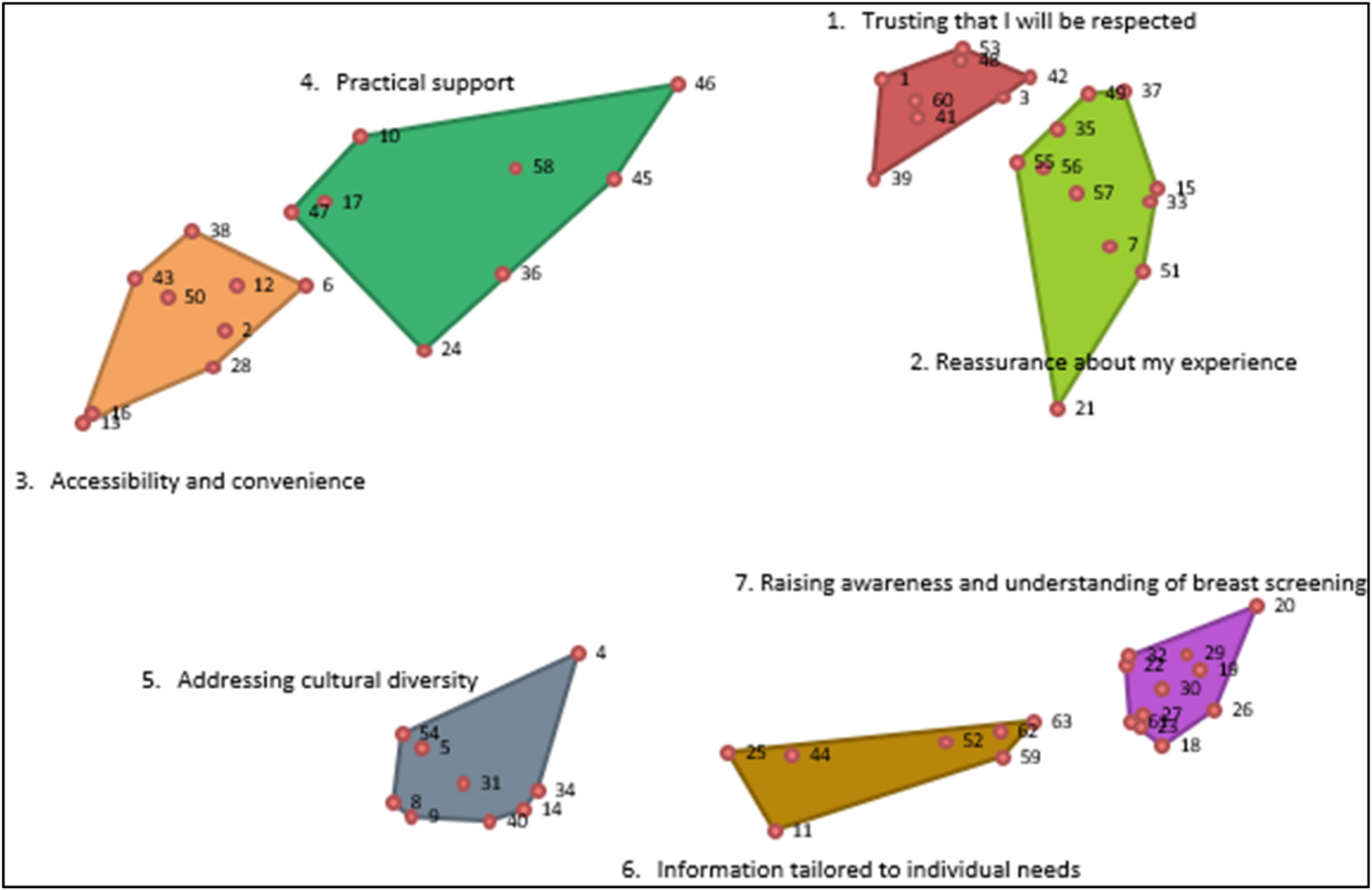
Seven‐cluster map with labels (each polygon represents a cluster/theme joining the outermost points in each cluster).

The cluster map comprised seven conceptually similar clusters (themes) from 63 statements (items of support) (Figure 2):

Cluster 1: Trusting that I will be respected (8 statements)

Cluster 2: Reassurance about my experience (11 statements)

Cluster 3: Accessibility and convenience (9 statements)

Cluster 4: Practical support (8 statements)

Cluster 5: Addressing cultural diversity (9 statements)

Cluster 6: Information tailored to individual needs (7 statements)

Cluster 7: Raising awareness and understanding of breast screening (11 statements)

The full list of statements in each cluster (theme) is available in Appendix 1. Cluster 2, ‘Reassurance about my experience’ and Cluster 7, ‘Raising awareness and understanding of breast screening’ had most statements (11), while Cluster 6, ‘Information tailored to individual needs’ had the least statements (7). The statements in each cluster (theme) were presented as points accompanied by corresponding statement numbers. The placement of a statement in a particular cluster was based on how participants grouped the statement. For example, statement 5, ‘Not feeling mistreated or judged due to my language barrier’, is positioned in Cluster 5, ‘Addressing cultural diversity’, because that is the cluster where participants most frequently placed the statement. The conceptual relationship between clusters (themes) is shown by the distance between them. The shorter the distance, the stronger the relationship. For example, Cluster 5, ‘Addressing cultural diversity’, is closer to Cluster 6, ‘Information tailored to individual needs’ than it is to other clusters (Figure 2).

**Figure 3:**
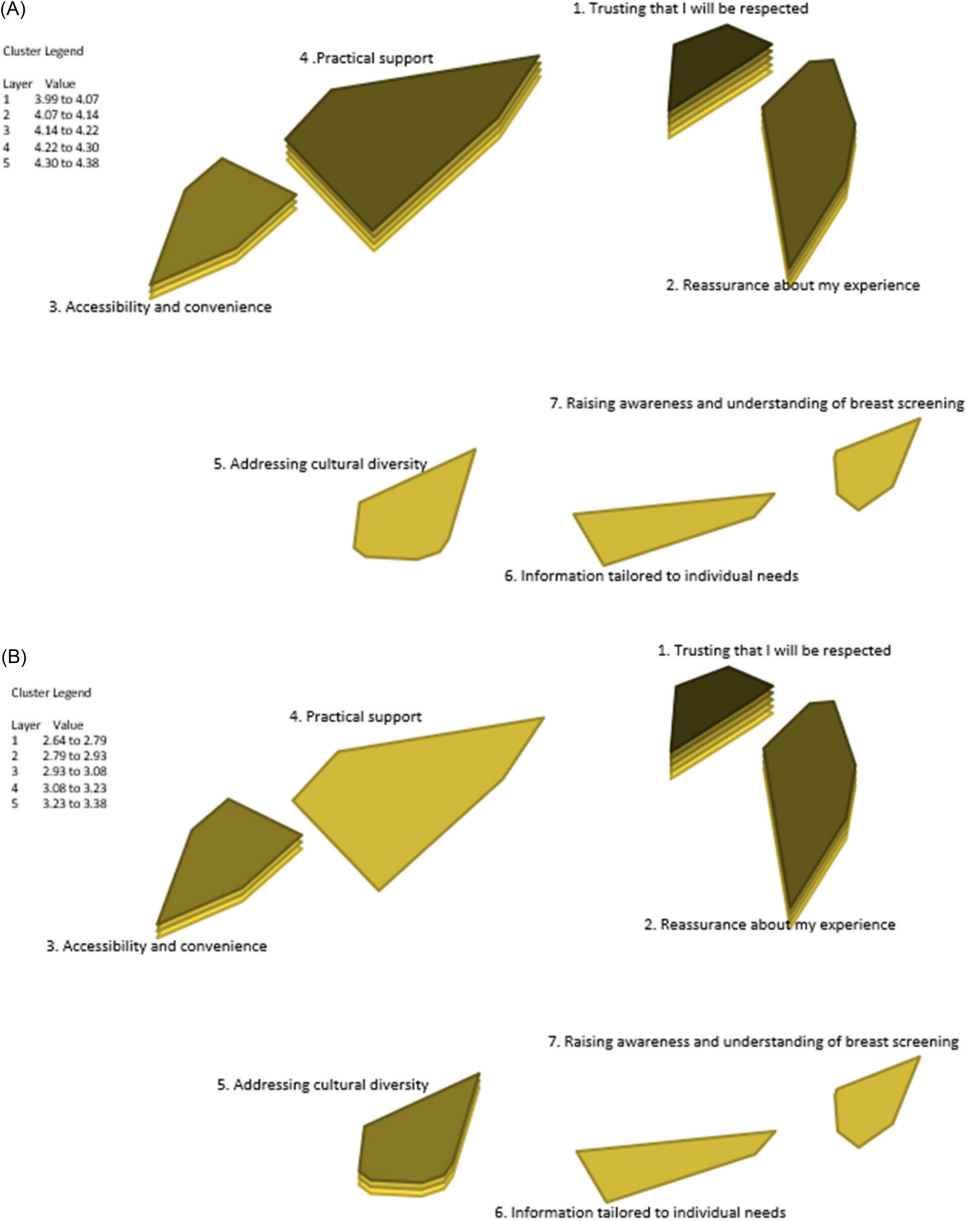
Cluster rating maps—importance (A) and accessibility (B).

The importance and accessibility (easy‐to‐get) of support at a cluster level are shown by the depth of the cluster polygons on the rating maps (Figure 3A,B, respectively). The deeper the polygon, the more important/accessible the support in the cluster. On average, Cluster 1, ‘Trusting that I will be respected’, was rated most important (mean = 4.38) and most easy to get (mean = 3.38). Cluster 7, ‘Raising awareness and understanding of breast screening’, was rated least important (mean = 3.99), while the support in Cluster 4, ‘Practical support’, was rated least easy to get (mean = 2.64).

There was general consistency between the two ratings for individual clusters (*r* = .58). However, some disparity was also found. For example, Cluster 4, ‘Practical support’ and Cluster 2, ‘Reassurance about my experience’ were rated higher for importance than for accessibility (*t* = 8.1365, *p* < .001; *t* = 7.4246, *p* < .001 respectively). In contrast, Cluster 5, ‘Addressing cultural diversity’ and Cluster 7, ‘Raising awareness and understanding of breast screening’ were rated lower for importance than for accessibility (*t* = 8.1325, *p* < .001; *t* = 9.0747, *p* < .001, respectively).

### 3.4 Go‐zone report

**Figure 4:**
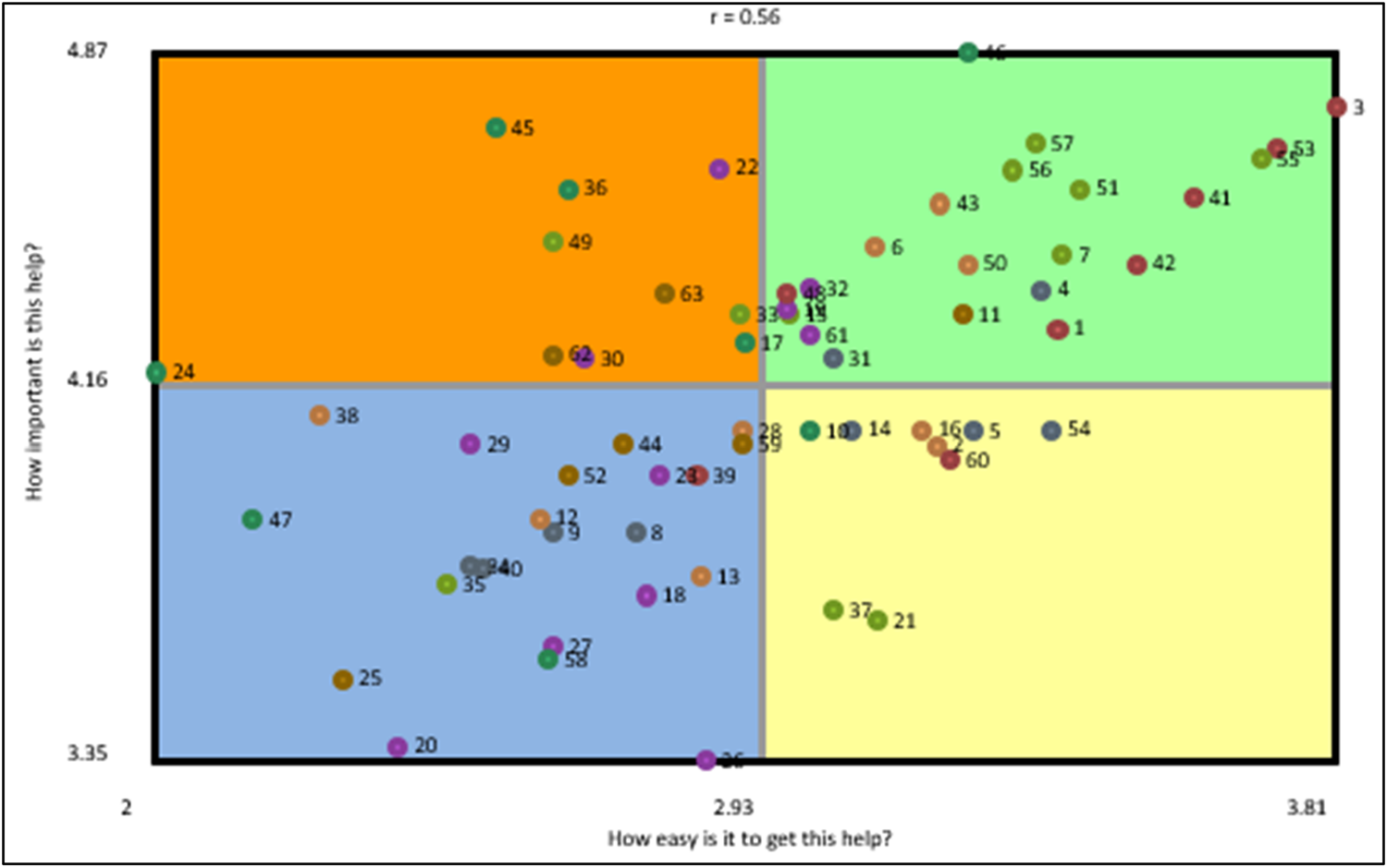
Go‐zone report of all statements showing two ratings (each point represents a statement accompanied by a corresponding statement number).

Presented in Figure 4 is the Go‐zone report for all statements (items), where statements were placed on a graph of four quadrants (each quadrant has its own colour), based on the mean rating of individual statements across the two rating variables: importance (mean = 4.16) and accessibility (mean = 2.93).

#### 3.4.1 Green zone

The green zone (top right) contains 22 statements, which were rated above the mean for both variables, showing support that was perceived as highly important and easy to get. Table 2 shows the top 10 statements in this zone. All seven clusters (themes) were represented within the statements in this zone. Most statements were from Cluster 1, ‘Trusting that I will be respected’ (6), and Cluster 2, ‘Reassurance about my experience’ (6).

**Table 2:** Top 10 statements in the green zone (top right) (important and relatively easy to get).

No	Statements	Mean rating for importance	Mean rating for accessibility (easy to get)	Mean rating	Ranking	Cluster
3	Respect for my dignity	4.76	3.81	4.28	1	1
53	Feeling I can trust the staff who carries out my screening	4.67	3.71	4.19	2	1
55	Feeling reassured that my screening appointment and results will be kept confidential	4.65	3.69	4.17	3	2
41	A female staff who carries out my screening	4.56	3.59	4.07	4	1
46	Knowing that I can go to my GP if I feel anything on my breast no matter what age I am	4.87	3.24	4.06	5	4
57	Being able to ask questions	4.68	3.34	4.01	6	2
51	Reassurance that breast screening can indicate early signs of breast cancer	4.58	3.41	4.00	7	2
56	Having my concerns about screening recognized and listened to	4.62	3.31	3.97	8	2
42	Knowing that my screening will be carried out by a female staff	4.42	3.50	3.96	9	1
7	Reassurance that breast screening test is safe	4.44	3.39	3.91	10	2

*Note*: Cluster 1: Trusting that I will be respected; Cluster 2: Reassurance about my experience; Cluster 4: Practical support.

Abbreviation: GP, general practitioner.

#### 3.4.2 Orange zone

The orange zone (top left) contains 10 statements, which were rated above the mean for importance, but below the mean for accessibility, showing support that was perceived as highly important, but relatively not easy to get (Table 3). The statements in this zone belonged to five clusters, and most statements were from Cluster 4, ‘Practical support’ (4).

**Table 3:** Statements in the orange zone (top left) (important and relatively not easy to get).

No	Statements (*n* = 10)	Mean rating for importance	Mean rating for accessibility (easy to get)	Mean rating	Ranking	Cluster
22	Information that would help me to check at home and understand any changes in my breast tissue	4.63	2.86	3.74	1	7
45	Quick access to screening if I feel a lump in my breast (two years ago, I felt a lump on my breast and my GP had referred me to have a breast screening at the hospital)	4.71	2.52	3.61	2	4
36	A new appointment letter if I have missed one for a reason, for example, due to ill health	4.58	2.63	3.61	3	4
33	Reassurance as I am too scared to go in case I get diagnosed with breast cancer	4.31	2.89	3.60	4	2
17	Having the screening service close by and easily accessible, for example, in my local supermarket or GP surgery	4.25	2.90	3.58	5	4
63	Health education programmes on breast screening that consider my level of health literacy	4.35	2.78	3.57	6	6
49	Improved X‐ray machine so that it doesn't hurt	4.47	2.61	3.54	7	2
30	Case studies of people who have had cancers detected early	4.22	2.66	3.44	8	7
62	More targeted information on breast cancer and breast screening services for women from different ethics groups	4.23	2.61	3.42	9	6
24	A one stop shop where all health screening (smears/mammogram etc) can be completed during one appointment	4.19	2.00	3.09	10	4

*Note*: Cluster 2: Reassurance about my experience; Cluster 4: Practical support; Cluster 6: Information tailored to individual needs; Cluster 7: Raising awareness and understanding of breast screening.

Abbreviation: GP, general practitioner.

#### 3.4.3 Yellow zone

The yellow zone (bottom right) contains nine statements, which were rated below the mean for importance, but above the mean for accessibility, showing support that was perceived as least important and easy to get. The statements in this zone belong to five clusters, while most statements were from Cluster 5, ‘Addressing cultural diversity’ (3).

#### 3.4.4 Blue zone

The blue zone (bottom left) contains 22 statements, which were rated below the mean for both variables, showing support that was perceived as least important and relatively not easy to get. All seven clusters were represented within the statements in this zone. Most statements were from Cluster 7, ‘Raising awareness and understanding of breast screening’ (7).

### 3.5 Pattern matching reports

**Figure 5:**
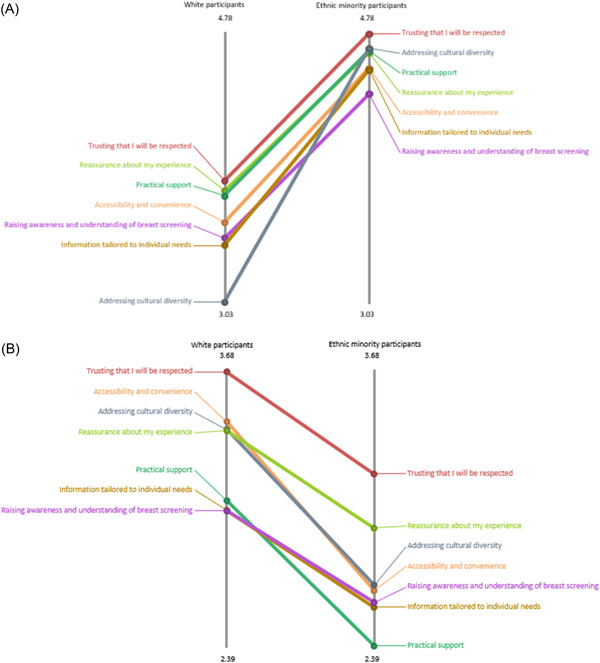
Rating on importance (A) and accessibility (B)—White participants versus ethnic minority participants.

**Figure 6:**
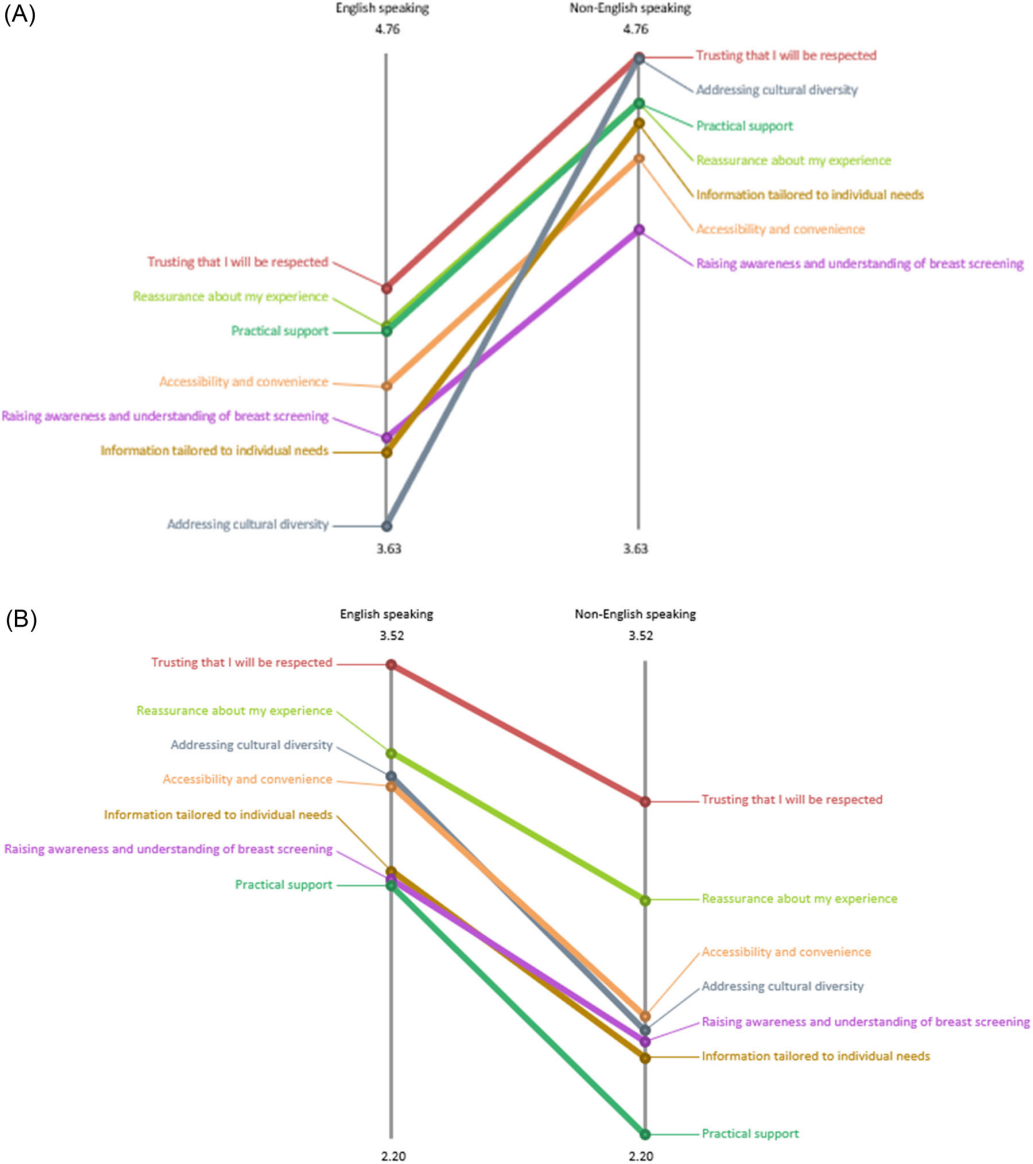
Rating on importance (A) and accessibility (B)—English‐speaking versus non‐English speaking participants.

**Figure 7:**
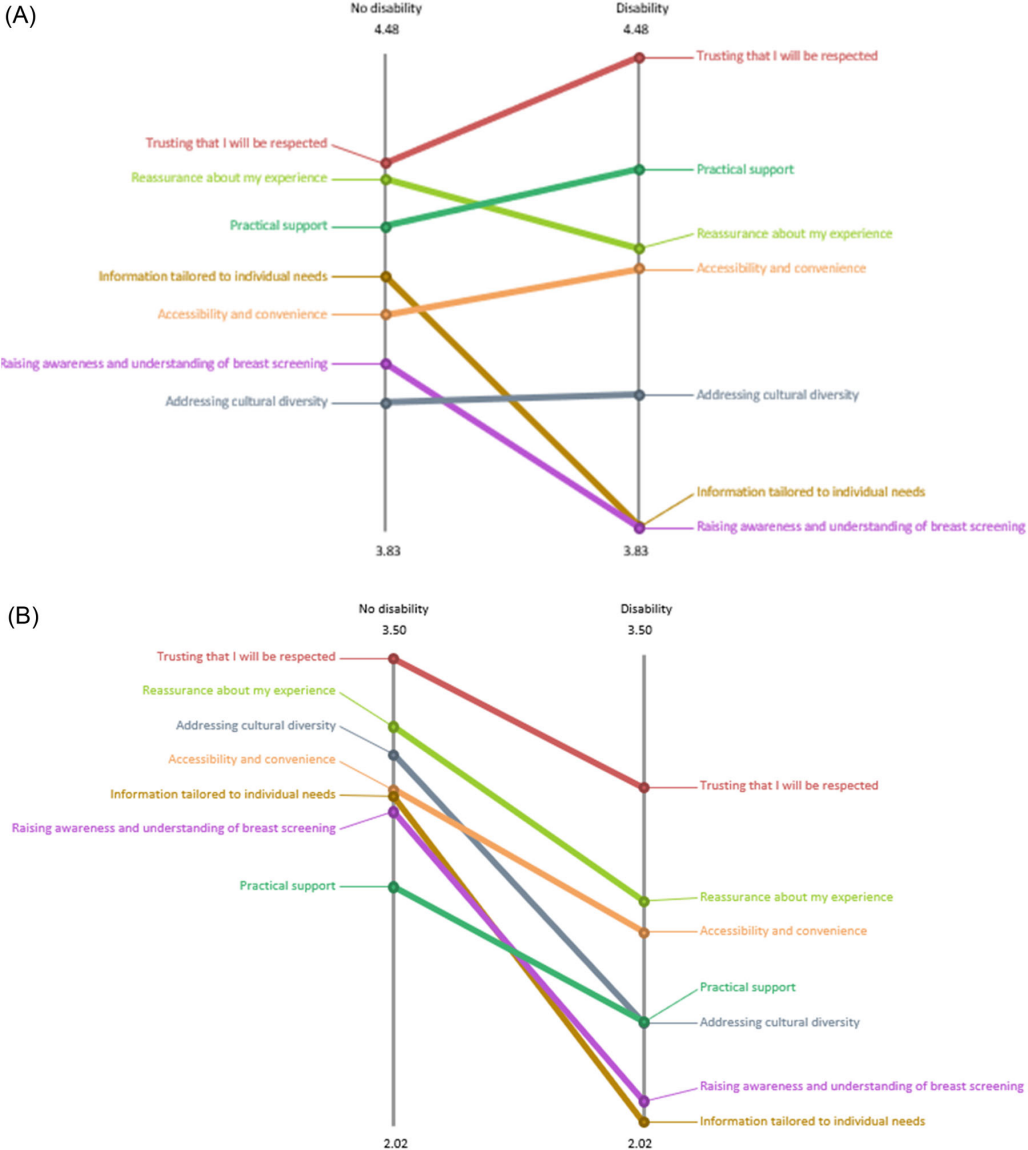
Rating on importance (A) and accessibility (B)—disability versus no disability participants.

Absolute pattern‐matching reports were produced to compare the two ratings based on ethnicity (Figure 5), language (Figure 6) and disability (Figure 7) at a cluster level.

Ethnic minority participants (*n* = 13) rated higher for importance (Figure 5A), but lower for accessibility (Figure 5B) for all clusters (themes), compared to White participants (*n* = 21). A similar pattern was found based on language. Non‐English speaking participants (*n* = 11) rated higher for importance (Figure 6A), but lower for accessibility (Figure 6B), compared to English‐speaking participants (*n* = 21). Figure 7 shows the disparity in responses based on disability. In terms of the importance rating, compared to participants with no reported disability (*n* = 23), participants with a disability (*n* = 11) rated higher on four clusters—Cluster 1, ‘Trusting that I will be respected’; Cluster 3, ‘Accessibility and convenience; Cluster 4 ‘Practical support’; Cluster 5, ‘Addressing cultural diversity’, but lower on three clusters – Cluster 2, ‘Reassurance about my experience’; Cluster 6, ‘Information tailored to individual needs’; Cluster 7, ‘Raising awareness and understanding of breast screening’ (Figure 7A). Furthermore, participants with a reported disability rated lower for accessibility in all seven clusters than those without disability (Figure 7B).

## 4 DISCUSSION

In this study, GCM was used to explore the support required to participate in breast screening from the perspectives of women residing in a GP cluster in Newport West, Wales. The results have outlined the importance and accessibility (easy to get) of a range of support, providing useful evidence to inform the development of future interventions.

The findings showed seven broad areas that participants required support in undertaking breast screening in relation to trust, experience, accessibility and convenience, practical support, cultural diversity, tailored information and awareness. These findings highlight the need for diverse practical, cultural and psychosocial support, and contribute to the wider literature where a range of barriers (personal, socioeconomic, psychological, cognitive and structural) to breast screening have been reported.^11^, ^12^, ^13^, ^14^, ^15^, ^16^

The support in the theme ‘Trusting I will be respected’ was rated most important and easy to get. These findings are encouraging as participants perceived that it was easy for them to get access to important support in this area. On the other hand, the theme ‘Practical support’ was rated the third most important, but least easy to get; most statements in the orange zone (rated most important but relatively not easy to get) were also related to the ‘Practical support’ cluster, such as being screened quickly if needed, timely reminders and convenient and accessible screening location. These findings suggest the need to consider the practicality for women and remove barriers to make screening more accessible. Previous research has put more emphasis on the need to enhance knowledge of breast cancer and awareness of breast screening, improve personal experience of breast screening, increase trust in health professionals delivering screening services, and reduce cultural, structural and organizational barriers to breast screening.^11^, ^12^, ^36^ However, our findings highlight the value of accessible support related to practicality.

Different perspectives were found based on participants' ethnicity and English‐speaking. Participants from ethnic minority backgrounds and those who did not speak/read English rated higher for importance, but lower for accessibility, than White participants and English‐speaking participants. These findings indicate that the main issues around low uptake of breast screening in women from ethnic minority backgrounds are not necessarily associated with the lack of awareness of breast cancer or breast screening, but with the lack of access to relevant support in undertaking screening. Similar findings have been reported by others.^14^, ^15^ Language needs, such as the need for interpreters and information provided in a person's first language, were identified among many other needs in this study but were not perceived as the most important. Nonetheless, issues around language barriers to navigating health care systems, including breast screening services, have been widely reported in the literature.^16^, ^17^ It is unlikely that women can make informed choices without a good understanding of breast screening, breast cancer, the procedure, and the ways in which to access screening services. However, narrowly focusing on language barriers would overlook the wider social, cultural, structural and organizational factors associated with breast screening inequalities.

Some disparity was found based on disability. For participants reporting a disability, being respected, reassurance about their experience, and practicality (e.g., accessible location and transport arrangements) were considered more important, but less easy to achieve, highlighting the importance of nonjudgmental attitudes of service providers and the need to improve access to facilities (e.g., a convenient screening location) and practical support (e.g., transport). These findings may provide evidence explaining the low attendance of breast screening in women with various disabilities in many countries,^37^, ^38^, ^39^, ^40^ and showing the support and resources women with disabilities may need to participate in breast screening.

### 4.1 Strengths and limitations

Due to the Covid‐19 pandemic, we initially planned to conduct the study solely online. However, this proved challenging for some women, for example, women who did not read/speak English, who were not IT competent, or who had no access to a computer. Therefore, data were also collected offline. This approach made our research more inclusive by engaging and hearing the views of many women who would have otherwise been excluded.

There are three main limitations. First, five local community members were recruited as co‐researchers to approach women in various ethnic minority communities. These co‐researchers were fluent in English, as well as in Bengali, Punjabi, Hindi, Urdu, Somali, Arabic or Yemeni. All study materials were orally translated, as the co‐researchers and participants were not necessarily able to read or write in the languages they spoke. This approach helped us reach women of ethnic minorities, while we relied on co‐researchers for information translation, meaning we had little control over the accuracy of translation.

Second, of the three activities (brainstorming, sorting and rating), fewer participants (*n* = 23) completed the sorting activity, as many participants, especially those who needed translation found it very challenging and time‐consuming. However, the number of participants who completed in each activity is in line with the recommended sample size for a GCM study.^29^

Third, as the work was commissioned by a GP cluster to understand the needs of the local population served by the cluster, we only recruited participants living in the areas covered by the cluster. We acknowledge that our sample is relatively small, self‐selected and not representative of women in the cluster or women in other regions of Wales or beyond due to the difficulties in obtaining a sample frame and in engaging women who did not usually attend breast screening. Therefore, our findings should be interpreted in this context. However, our sample size was reasonable for a GCM study, and the stress value of this study (measuring validity) is also within the recommended range.^29^, ^34^ Furthermore, our flexible use of GCM (and its participatory approach) enabled us to engage, involve and hear from women from seven ethnic groups, and our findings offer valuable direction for future interventions to improve the uptake of breast cancer screening services among these groups.

### 4.2 Recommendations

Based on the findings, four main recommendations are made. First, understanding the support women require to undertake breast screening is the initial step to tackling screening inequalities. As found in this study, practical support related to practicality was highly valued by participants but felt not easy to access, while raising awareness was viewed as less important. Therefore, while maintaining awareness raising, future interventions should also focus on providing more accessible practical support, such as support relating to screening location and transport. An exploration of what support works for whom, in what circumstance, and in what context is needed, which takes into account individual needs and the demographics of a local population, rather than a ‘one size fits all’ approach.

Second, findings presented in the Go‐zone report can inform future intervention development or decision‐making for commissioning support services to increase breast screening uptake. Statements (items) presented in each quadrant need to be examined carefully against current breast screening services in an area. For example, more attention may be paid to the type of support presented in the orange zone (highly important, but less easy to get) by developing additional services or improving existing services. However, efforts also need to be made to maintain the type of support seen as important and easy to access (green zone).

Third, the seven clusters (themes) identified in this study can be used as a broad topic guide when developing future staff training programmes, organizational policy or assessing the competencies of health professionals providing breast screening services.

Lastly, considering the issues around accessible support, especially among participants who do not speak or read English and/or have disabilities, future work needs to focus on improving access to support tailored to individual needs, in particular, the type of support rated highly important, but not easy to get, such as practical support (e.g., transport and accessible screening locations).

## 5 CONCLUSION

This is the first study using GCM as a consensus approach to explore the importance and accessibility of support from the perspectives of women eligible but not necessarily attending the screening, in Wales. The range of support identified by participants in this study can inform the development of interventions to improve breast screening uptake in a local context by removing personal, cultural, structural and organizational barriers to accessing screening. A combination of online and offline mechanisms has allowed a range of women to have a voice and contribute to this research. Gaining views from a range of women, especially those who do not speak English, remains important in developing services for an ethnically diverse population. Future research is still needed by engaging a larger and more representative sample to identify and address any ongoing issues around participating in breast screening.

## CONFLICT OF INTEREST STATEMENT

The authors declare no conflict of interest.

## ETHICS STATEMENT

Ethical approval was granted by the Faculty of Life Sciences and Education Research Ethics Committee at the University of South Wales (201204HR) and the Central Bristol Research and Ethics Committee (21/SW/0028). All participants gave written consent.

## Data Availability

Due to the nature of this research, participants of this study did not agree for their data to be shared publicly, so supporting data is not available. The data that support the findings of this study are available from the corresponding author upon reasonable request.

## APPENDIX 1

Clusters and statements

**Cluster 1—Trusting that I will be respected**

**Statements (*n*** = **8)**

1. No rush to get undressed and dress back due to disability

3. Respect for my dignity

39. If something could help with the pain

41. A female staff who carries out my screening

42. Knowing that my screening will be carried out by a female staff

48. Making breast screening comfortable

53. Feeling I can trust the staff who carries out my screening

60. Knowing that the staff who carries out my screening is aware of my conditions, such as my learning disability

**Cluster 2—Reassurance about my experience**

**Statements (*n*** = **11)**

7. Reassurance that breast screening test is safe

15. Reassurance that the scan doesn't hurt (as many women say it's painful)

21. Knowing who is providing the breast screening service, for example, doctors or national breast screening service

33. Reassurance as I am too scared to go in case I get diagnosed with breast cancer

35. An adult to be with me for reassurance to avoid getting distressed

37. If it were less embarrassing

49. Improved X‐ray machine so that it doesn't hurt

51. Reassurance that breast screening can indicate early signs of breast cancer

55. Feeling reassured that my screening appointment and results will be kept confidential

56. Having my concerns about screening recognized and listened to

57. Being able to ask questions

**Cluster 3—Accessibility and convenience**

**Statements (*n*** = **9)**

2. Longer time for mobile screening vehicle to be at one sit

6. Disabled access to mobile screening facility

12. Breast screening test to be taken close to my house due to my disability

13. Reminder message sent via email

16. Receiving a text message for my appointment and a reminder

28. Slick appointment system

38. Walk in screening clinic for locals who missed their appointments

43. Timely appointment system

50. Convenient date and time for a screening appointment

**Cluster 4—Practical support**

**Statements (*n*** = **8)**

10. Reassurance that I will be seen on time

17. Having the screening service close by and easily accessible, for example, in my local supermarket or GP surgery

24. A one stop shop where all health screening (smears/mammogram etc) can be completed during one appointment

36. A new appointment letter if I have missed one for a reason, for example, due to ill health

45. Quick access to screening if I feel a lump in my breast (two years ago, I felt a lump on my breast and my GP had referred me to have a breast screening at the hospital)

46. Knowing that I can go to my GP if I feel anything on my breast no matter what age I am

47. Transport provided if needed

58. Having someone to look after the people I care for when I go for screening

**Cluster 5—Addressing cultural diversity**

**Statements (*n*** = **9)**

4. Not feeling mistreated or judged due to my background

5. Not feeling mistreated or judged due to my language barrier

8. Phone call from my clinic or breast screening services in my first language via an interpreter

9. Invite letter written in my first language as I can't read English

14. Easy‐to‐read English without medical terminology that I can't understand

31. A brief and clear appointment letter in simple English

34. Language support as I can hardly understand any English

40. An interpreter of my language

54. Knowing that the staff who carries out my screening is sensitive to my cultural beliefs

**Cluster 6—Information tailored to individual needs**

**Statements (*n*** = **7)**

11. Information in plain English that helps me understand how important breast screening is

25. Working with religious leaders as some religions may consider breast screening a taboo subject

44. Information about breast screening that is easy for me to understand and is provided in my language

52. Information about breast screening that is displayed/shared within my community, for example, community groups, churches and other faith settings etc.

59. Information about screening provided in different formats, such as hard copies, text messages, as well as online

62. More targeted information on breast cancer and breast screening services for women from different ethics groups

63. Health education programmes on breast screening that consider my level of health literacy

**Cluster 7—Raising awareness and understanding of breast screening**

**Statements (*n*** = **11)**

18. More information about breast screening in magazines or press

19. Good news stories of people who have survived because of breast screening

20. A cartoon type video showing the actual process of breast screening for reassurance

22. Information that would help me to check at home and understand any changes in my breast tissue

23. More information and education for younger people

26. Reiterating the importance of breast screening during my antenatal classes

27. Educational programmes in schools for boys and girls

29. Case studies of people who wish they'd have gone to breast screening but didn't and their breast cancer (which could have been cured) is now advanced

30. Case studies of people who have had cancers detected early

32. Information that helps me understand how breast screening can help me

61. Information about breast screening available from multiple sources, such as newspapers, exhibitions, lectures, information stalls and posters
